# Can we improve healthcare with centralized management systems, supported by information technology, predictive analytics, and real-time data?: A review

**DOI:** 10.1097/MD.0000000000035769

**Published:** 2023-11-10

**Authors:** Liza Grosman-Rimon, Donny H.Y. Li, Barabra E. Collins, Pete Wegier

**Affiliations:** a Research Institute, Humber River Health, Toronto, Ontario, Canada; b University of Toronto, Institute of Health Policy, Management and Evaluation, Ontario, Canada; c McMaster University, Faculty of Health Sciences, Hamilton, Ontario, Canada.

**Keywords:** centralized management systems, predictive analytics, real-time data, supported by information technology

## Abstract

This narrative review discusses the effects of implementing command centers, centralized management systems, supported by information technology, predictive analytics, and real-time data, as well as small-scale centralized operating systems, on patient outcomes, operation, care delivery, and resource utilization. Implementations of command centers and small-scale centralized operating systems have led to improvement in 3 areas: integration of both multiple services into the day-to-day operation, communication and coordination, and employment of prediction and early warning system. Additional studies are required to understand the full impact of command centers on the healthcare system.

## 1. Introduction

Healthcare organizations around the world continuously strive to be high-reliability organizations with consistent, predictable, safe, and effective operations, while managing high volumes of patients, within complex healthcare systems, and with varying levels of resources.^[1]^ Despite efforts to improve the quality of healthcare delivery while balancing resource utilization, many patients still suffer from preventable harms and resources often go to waste.^[2–4]^ Many of the challenges faced by healthcare organizations stem from capacity constraints and an increasing demand to provide efficient services.^[5]^ These problems are evident in hospital-associated infections,^[6]^ sub-optimal medical management and medication errors,^[7]^ failures to coordinate care,^[8]^ and harmful events during transition and discharge,^[9]^ all of which can result in related adverse events, including morbidity and death. Further, systematic mismatches in resource supply and demand across a hospital may lead to strains, most notable in the emergency department, post-anesthesia care, intensive care units, and patient admissions.^[10]^

While hospitals are struggling, other industries which operate under hazardous and constrained conditions—space, aviation, traffic control, and the energy sector, including nuclear power, chemical, and oil and gas have successfully achieved high-reliability and exemplary standards of performance and safety.^[1,2,5]^ These industries have been aided in this through the development of data-driven command centers, centralized management systems which are supported by information technology, predictive analytics, and real-time data. These systems are critical to improving organizational communication, coordination, and accountability, as well as achieving high performance.^[10]^ Command centers rely on best-practices such as team co-location, automated real-time data collection, user-interfaces which provide a global view, predictive analytics, and clear protocols to proactively manage operations.^[11]^

These elements of high reliability including performance consistency allowing for long-lasting high levels of safety and exceptional operations should be adopted by our complex and overloaded healthcare systems to achieve predictable, safe, and effective operations. Although elements of high-reliability centers may be present in most healthcare systems,^[5]^ their application is usually fragmented and used in isolation, which may lead to sub-optimal outcomes, or in the worst case, to dysfunction.^[12]^

## 2. Smaller scale centralized operating systems

Several facets of these larger command center models have previously been implemented in healthcare as smaller-scale centralized operating systems and have focused on integration of multiple services into the day-to-day operation, improved communication and coordination, and employment of prediction and early warning systems. Studies have demonstrated that system which focus on these 3 facets have led to improvements in many aspects of healthcare delivery.

### 2.1. Integration of multiple services into the day-to-day operation

The integration of multiple services in smaller scale centralized operating systems has been shown to improve areas such as patient boarding and management, ambulance diversion, environmental service response time, as well as reduction in the time to bed assignment and turnover.^[10,13,14]^ One system to manage patient flow in a 3-campus academic health system integrated patient management, bed management, case management, environmental services, patient transport, and ambulance helicopter dispatches, into a single technology platform, which led to improvements of operations in the emergency department in the first year.^[10]^

A second implementation combined the teams responsible for bed management, transfers, internal patient transport, environmental services, and air/ground ambulance communications, into contiguous resulted in increased bed availability and decreased length of stay in the intensive care units, full utilization of capacity and services at partner hospitals, and maintained emergency department boarding times despite significant increases in emergency department demand.^[5]^

A fully integrated emergency department information system and process redesign with patient tracking, computerized charting and order entry, and direct access to patient historical data from the hospital data repository, resulted in increased clinical information available at the bedside and improvements to departmental workflow.^[15]^ Specifically, improvements included decreased length of stay by 1.94 hours, from 6.69 pre-intervention to 4.75 post-intervention; doctor-to-disposition time decreased by 1.90 hours, from 3.64 to 1.74; time from triage to first encounter with a doctor decreased by 0.54 hours, from 1.22 to 0.68; times for X-rays decreased by 0.18 hours from 0.92 to 0.74, and computerized tomography decreased by 1.56 hours, from 3.89 to 2.33; and lab turnaround time decreased by 0.59 hours, from 2.03 to 1.44.

### 2.2. Improved communication and coordination in smaller scale centralized operating systems

Smaller scale centralized operating systems contribute to optimization of operation in hospitals through the improvement of communication and coordination. Original work in this area was based on the hospital emergency incident command systems, used for control,^[13,16–18]^ coordination, and communication and management of events such as biocontainment,^[14,16]^ chemicals, biological, radiological, nuclear^[18]^ and natural disaster emergencies. Notably, the structure of the hospital emergency incident command systems does not rely on specific individuals, as it is flexible and expandable^[19]^ and facilitates communication during event management.^[13]^ Other early systems included an internet-based bed management system which was found to successfully expedite the direct admission of patients and keeping the emergency department off diversionary status, while increasing transfer requests by 48%, and decreasing denials due to a lack of capacity by 54%.^[20]^

More recent systems have included an electronic bed management system designed for a high degree of communication and coordination, which reported improvements in patient flow, patient experience, and bed turnover time, with reduction in the mean bed turnover time from 111 minutes to 49 minutes.^[21]^ A combined communications center and transfer service, supported through electronic throughput and flow centralized software, aimed for seamless entry of patients into the health system, coordination of the safest, most appropriate transport of patients, and efficient management of hospital throughput needs.^[22]^ This resulted in improvements in throughput, with 10% average increase in the total number of internally supported ambulance discharges, enhanced accountability by providing real-time data to all nursing unit leaders, improvement in internal bed transfer assignment times with a 5% increase in the number of bed placements.

### 2.3. Employment of prediction and early warning systems

Prediction of future conditions and activation of early warning systems is third key facet of the command center approach. These systems allow for timely recognition of warning signs from deteriorating patients and alerting of life-threatening conditions, and consequently for the provision of appropriate treatment.^[23]^ However, the majority of these automated clinical deterioration detection systems to date have typically only been implemented in specific clinical areas without larger-scale integration and implementation. A range of studies have found that early warning systems assist in the identification of patient deterioration and mortality as well as sepsis risk.

#### 2.3.1. Deterioration and mortality.

The implementation of prediction and early warning systems, such as the rapid response system and real-time automated clinical deterioration alerts in some general medicine units has resulted in a lower rate of hospital mortality, cardiopulmonary arrests, and length of hospitalization.^[24]^ Although an increase was observed in the year-to-year number of rapid response activations, the study reported a decrease of 3.4 cardiopulmonary arrests per study year increment and a 0.08 day decrease in median hospital length of stay per study year increment. One hospital implemented an automated early warning score system which notified the medical emergency team scores above a pre-set threshold were observed.^[25]^ Those flagged patients saw significant reductions in the length of time from deterioration to medical emergency team activation (from a mean of 60 minutes to 34 minutes), admission rates to the intensive care unit were reduced (from 71.8% to 41.2%), and mortality significantly decreased (from 38.5% to 27.2%). Another early warning system for clinical deterioration in hospital resulted in increased medical emergency team alerts activation per 1000 admissions from 14.4 to 26.3, decreased in-hospital mortality per 1000 admissions from 15.1 to 12.9.^[26]^ A large implementation of a prediction and early warning systems in 19 hospitals was used to trigger interventions by rapid-response teams of patients at high risk of in-hospital deterioration, and was associated with decreased mortality 30 days after a trigger, translating to 3.0 deaths (95% Confident interval [CI], 1.2–4.8) avoided per 1000 eligible patients.^[23]^ Finally, a vital sign monitoring system signaling clinical deterioration in ward patients showed a significant reduction in cardiac arrests from 14 to 2 and in-hospital mortality from 173 to 147 events, over the study period.^[27]^

Other early warning systems have resulted in significant reductions in cardiac arrests.^[28,29]^ An early warning system using an algorithm was developed to detect early indicators of health decline that could cause cardiac arrest. This system drove a set of guidelines, assisting the critical care teams to manage patients before code blue is activated and following the deployment of the early warning system algorithm, the number of code blues declined by 11%.^[28]^ The implementation of a decision support system with automated screening for abnormal vital signs was associated with improved timeliness of hospital-wide rapid response system activations and reduced in-hospital resuscitations and mortality.^[30]^ Specifically, the interval between admission and first rapid response team activation time was lower in the decision support system group versus the conventional group (6.9 vs 9.8 days); cardiopulmonary resuscitation rate was lower (0.98 vs 1.35); length of hospitalization was shorter (23.3 vs 28.9); and in-hospital death rate was lower (15.0 vs 19.6), respectively.

An early warning score to improve the early identification of deteriorating hematologyoncology patients designed to prevent the development of critical illness and to facilitate timely intensive care unit (ICU) transfers revealed a significant in cardiopulmonary arrest codes by nearly 50%, while ICU transfers remained stable.^[29]^ An automated surveillance and alerting system in combination with a labor and delivery unit of nursing-driven early warning system improve detection of severely morbid postpartum hemorrhage after delivery.^[31]^

These early warning systems for mortality have been also used as prospective triggers for palliative care interventions. The Hospital-patient One-year Mortality Risk (HOMR) score has been implemented as a way to prospectively identify patients who are at high risk of mortality in the year following admission to hospital and who may benefit from a palliative approach to their care.^[32,33]^

#### 2.3.2. Sepsis risk.

One system increased early appropriate therapeutic and diagnostic interventions among non-intensive care patients at risk for sepsis.^[34]^ Specifically, patients in the intervention group, flagged by the early warning system, were more likely to have received at least 1 medical intervention for sepsis, compared with patients in the control group (70.8% vs 55.8%). Significant increases were also seen in antibiotic escalation (36.0% vs 23.8%), intravenous fluid administration (38.2% vs 23.8%), and oxygen therapy (20.2% vs 8.3%), were observed in the intervention compared with the control group, respectively. However, both groups had similar rates of ICU transfer, hospital mortality, hospital length of stay.

Another early warning system increased identification for sepsis in at-risk patients from 3.5% pre-implementation to 3.8% post-implementation and resulted in increases in early sepsis care, ICU transfer, and system activations for the general medicine units, while a trend was observed in decreased sepsis mortality and increased discharge from hospital.^[35]^

#### 2.3.3. Limitations.

In contrast, a handful of studies of these smaller scale centralized operating systems did not demonstrate clinical benefits. These included studies in which there was no observed reduction in ICU transfers, hospital mortality, or the need for post-discharge long-term care^[36]^; and no significant decrease in mortality, hospital length of stay, or ICU readmissions.^[37]^ One review focused on specific clinical events such as hospital infections, reported that sensitivity and specificity measures varied across studies.^[38]^ The investigators suggested that automated deterioration detection using electronic medical record data may be an important aid in caring for intensive care unit patients, but its usefulness is limited by variable electronic medical record detection approaches and performance.

## 3. A command center in the hospital setting

Hospitals around the world have been striving to improve healthcare quality, drive efficiencies, and reduce operating expenses.^[10]^ Yet, larger scale command centers, which centralize all care functions and integrate with all divisions and departments within the organization have only been implemented in a handful of medical systems around the world.

Hospital-based command centers optimizes patient-care and hospital resource utilization by employing information technology systems to conduct 24/7 monitoring and allowing for accelerated clinical and administrative decision-making. They collect real-time information from multiple hospital systems on areas including patient management, risk of harm, patient deterioration alerts, beds, patient transfers, consultations, admissions, discharges, and other aspects of care.^[12,39]^ This data is then displayed on large screens, known as “tiles,” which include analytical functions to facilitate appropriate clinical interventions. While hospital-based command centers are becoming more common, the published literature is still sparse.

The first data-driven command center was launched in 2016 at Johns Hopkins Hospital and led to a 30% reduction in the number of emergency patients who had to wait for an inpatient bed and a 1-hour reduction in the time it took to retrieve data and identify patients for transfer into the Johns Hopkins facility.^[39]^ Additionally, the creation of this command center led to an increase in occupancy, from 85% to 92%, while boarding of emergency department patients in medicine beds decreased from 9.7 hours to 6.3 hours.^[11]^

In 2017, Humber River Hospital became the second hospital in North America to implement a command center for managing patient access and flow in real-time with predictive analytics, which included early identification of patients at risk of harm and deterioration.^[12]^ These innovations have resulted in a significantly lower overall hospital harm rate per 100 patients, compared to all other hospitals in the same province (2.2 vs 5.7). The hospital harm rate was also significantly lower at Humber River Hospital, compared to all other hospitals in the province, for medication conditions (1.0 vs 3.3), infection conditions (0.5 vs 1.9), patient accidents (0.1 vs 0.2), and associated procedures (0.8 vs 1.3).^[40]^ These rates have dropped significantly over time after the implementation of the command center, while province-wide statistics have remained flat.^[40]^ A third implementation at Jefferson Hospital revealed their command center led to significant decreases in ambulance diversions per month (from 86 to 7), time from entry into the emergency department door seeing a to provider (from 74 to 41 minutes), environmental services turnaround time (from 115 to 72 minutes), and bed request to assign time (from 153 to 105).^[10]^

Similarly, the command center at the Carilion Clinic significantly improved operational performance across their hospital network in 4 key areas: transfer volume; emergency department boarding times; ICU bed availability; and balancing patient transfers throughout the network of hospitals.^[5]^ Results included increases of 19% in patient transfer volumes and 7% in emergency department admission volume, and a decrease in length of stay by 0.3 days. These areas of improvements were attributed to increased situational awareness the command center provided, which allowed for real-time changes to process as required.

Other healthcare centers around the world have launched or are in the process of developing command centers with the goals of improving patient outcomes, maximizing allocation of resources, operational efficiency, and reducing operating costs. While the initial evidence has demonstrated that meaningful large-scale improvements to healthcare delivery are possible with the implementation of hospital-based command centers, the current literature remains sparse on effectiveness, quality of care, and safety.^[41]^ Future studies should investigate the effects of commend center implementation on clinical decisions making, patients management, and clinical outcomes.

## 4. Discussion

Globally, healthcare organizations, which are challenged by the growing number of aging populations, the burden of chronic diseases, and rising healthcare costs are seeking to provide innovative models of healthcare delivery.^[42]^ Transforming the hospitals into high-reliability organizations with a command center has the potential to improve many aspects of the healthcare system.^[1]^ Indeed, command centers are centralized management systems which are supported by information technology, predictive analytics, and real-time data which has a potential to improve the health care system. Integrating elements of high reliability in the design of command centers includes performance consistency, that yields predictable, safe, and effective operations. Furthermore, team co-location, and tiles, which provide a global view, in combination with predictive analytics, and clear protocols allow for a proactive management of operations.^[11]^ These systems integrate multiple services into the day-to-day operation^[13,14,16,17]^ and improve communication and coordination^[13,16–18,20–22]^ as well as employment of prediction and early warning systems.^[23–31]^

Although the command center is a centralized management system, it allows for both centralized model of care by providing care in resource intensive institutions such as hospitals and healthcare center, as well as de-centralization, providing care in the community or supporting patients’ self-management at home. While currently the majority of the research to date focuses on the centralized model of care, the command center has the potential to support also home-based/community-based healthcare models. For example, home-based mental health therapies operating on low-cost technologies^[43]^ can be integrated into the command operation healthcare for remote monitoring and self-management. Integration of artificial intelligence (AI) technologies into the command center operation can improve healthcare systems performance during the next pandemic to improve vaccine production and supply chains as well as navigate the complexity of the pandemic.^[44]^ However, with advancements in digital health, internet-connected health systems and devices, in both centralized and de- centralized models of care, cyber-risk assessments and cyber-security should be implemented in order to preserve patients’ data security, privacy and trust.^[45]^ In both centralized and de-centralized model of care, the integration of AI as part of the command center could be valuable for delivering effective and high-quality care,^[42]^ including the improvement of safety in the health care system.^[46]^ Future studies should examine the usefulness of AI in the elimination of the “risk of harm” and “never events” in the context of a commend center.

**Figure 1.**
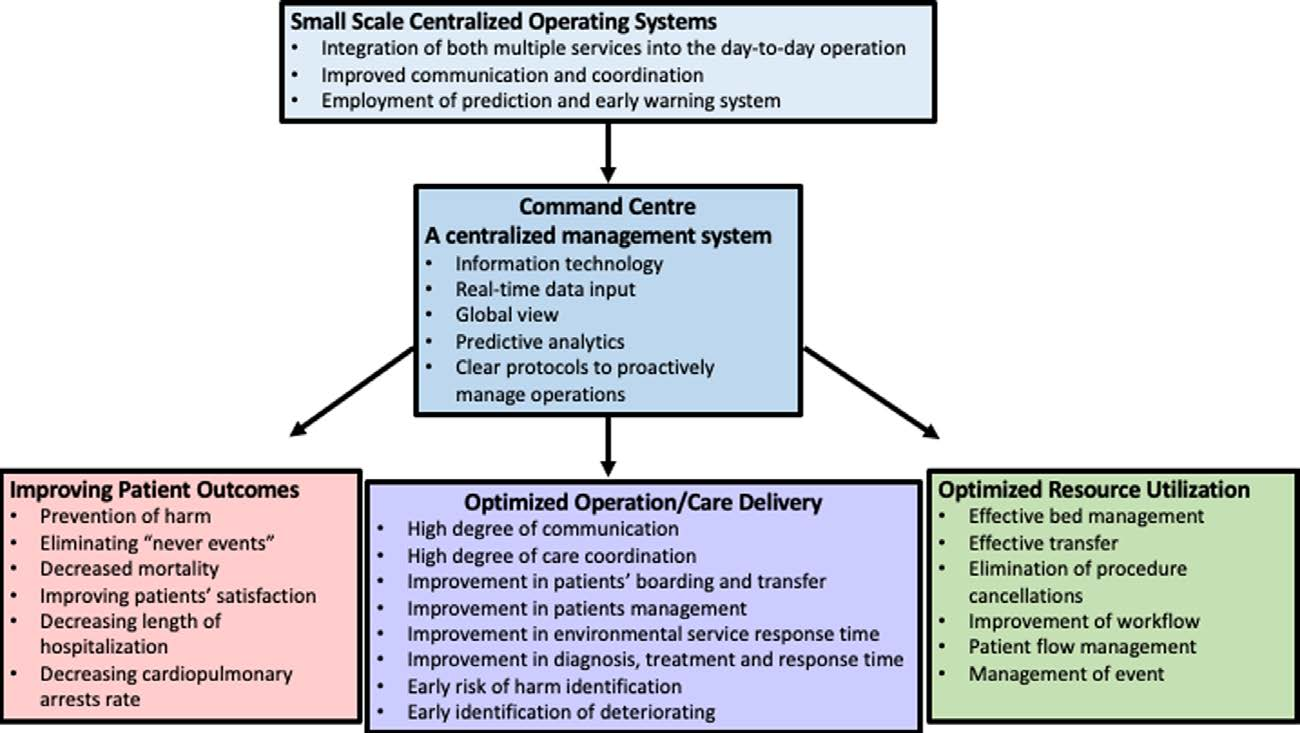
The effects of commend center implementation on patient outcomes, operation/care delivery, and resource utilization.

Several main areas of improvements were identified following the implementation of smaller scale centralized operating systems, including optimized resource utilization, optimized operation/care delivery, and improving patient outcomes (Fig. 1). Optimized resource utilization included effective bed management, effective transfer, elimination of procedure cancelation, improvement of workflow, and patient-flow management, and management of event.^[5,15,20,21]^ Furthermore, the optimization of operation and care delivery in smaller scale centralized operating systems included a high degree of communication, a high degree of care coordination, improvements in environmental service response times, patients’ boarding and transfer, patients’ management, diagnosis, treatment and response time, as well as early identification of risk of harm and deterioration.^[10,13–18,22,29–35]^ Improving patient outcomes in smaller scale centralized operating systems included prevention of harm, eliminating “never events,” reducing mortality and length of hospitalization, decreasing cardiopulmonary arrests rate, and improving patients’ satisfaction.^[23–30,35]^ While smaller scale centralized operating systems were investigated extensively, command centers are relatively new in the healthcare system. Most of these studies showed optimized resource utilization,^[5,10,11]^ optimized operation/care delivery,^[5,10]^ and only 1 showed an improvement in patient outcomes.^[12]^ More studies are required to investigate the effects of command centers on patients’ related outcomes.

There are several recommendations when implementing and launching a command center.

## 5. Recommendations for future work

- A hospital-based command center acts as a “brain” overseeing the entire hospital operation, optimizing patient flow, delivery of care, and clinical outcomes. However, the implementation should be tapered, with a gradual employment of the system in a few departments at the initial stage based on priority, with the goal of managing operations in all hospital departments.
- During the development of command center tiles, clinicians and other key stakeholders should work in partnership and collaborate to optimize operation.
- Continuous improvement is an important part of the development and maintenance of a command center, requiring a highly detailed and iterative process which includes monitoring, evaluation, and adaptation while engaging with clinicians and other key stakeholders.
- Rigorous, holistic evaluations of the impacts of the command center must be planned prior to the implementation of a command center to ensure all data points necessary to evaluate the command center are identified and gathered. This should include detailed recording of how the implementation of the command center changed clinical workflows and the staff involved in those workflows, in addition to the patient and corporate outcomes of the implementation.

## 6. Conclusion

The initial results from the handful of studies that assessed the outcomes of hospital-based command center implementations, combined with the work on smaller scale centralized operating systems—has provided strong support for a centralized management system which combines information technology with real-time data can improve the quality of the healthcare system, including operations, clinical decisions making, patients management, as well as optimizing clinical outcomes.(Table 1) However, the current literature remains sparse and additional studies are required. The full impact of command centers on the healthcare system will be revealed in future studies and will allow us to determine whether patient safety has improved and “risk of harm” and “never events” were eliminated, as well as whether hospital operation and use of resources were optimal.

**Table 1:** Summary of results.

Year	Author	Location	Setting	Outcomes
Small Scale Centralized Operating Systems—Integration				
2010	Baumlin, K	Mount Sinai Medical Centre, New York, USA	Hospital (Emergency Department)	Post-intervention of an emergency department information system compared to pre-intervention:
				↓ Average ED length of stay from 6.69 to 4.75 h
				↓ Doctor-to-disposition time from 3.64 to 1.74 h
				↓ Triage to first-to-doctor time from 1.22 to 0.68 h
				↓ X-ray turnaround time from 0.92 to 0.74 h
				↓ CT scan turnaround time from 3.89 to 2.33 h
				↓ Lab turnaround time from 2.03 to 1.44 h
Small Scale Centralized Operating Systems—Communication and Coordination				
2005	Hemphill, R	Saint Francis Hospital, Oklahoma, USA	Hospital (Emergency Department)	Post-intervention of a bed management Access Centre compared to pre-intervention:
				↑ Expedition of direct admission of patients
				↑ Transfer requests by 48%
				↓ Denials due to “no capacity” by 54%
2015	Morris, M	Carilion Clinic, Virginia, USA	Hospital (Emergency Department)	Post-intervention of a central transfer and communications center compared to pre-intervention:
				↑ Satisfaction, accountability, internal bed assignment times
				↑ Internally supported ambulance discharge by 10%
				↑ Patients moved internally to clean bed by 5% when assigned goal time of under 1 h
2013	Tortorella, F	Anderson Cancer Centre, Texas, USA	Hospital	Post-intervention of a bed management system compared to pre-intervention:
				↑ Patient flow, patient experience, bed turnover time
				↓ Time of room being notified as dirty, to cleaned and ready, from 63 to 49 min
				↓ Bed turnover time from 111 to 49 min
Small Scale Centralized Operating Systems—Early Warning and Prediction				
2020	Escobar, GJ	KPNC Hospital System, USA	Hospital (Non-ICU)	↓ Mortality by 3 deaths avoided per 1000 eligible patients per year following intervention of an automated predictive model identifying high-risk patients
2022	Jerng, JS	National Taiwan University Hospital, Taiwan	Hospital (General Ward)	Decision support system group compared to conventional group:
				↓ Interval between admission and first rapid response activation (6.9 vs 9.8 days)
				↓ Cardiopulmonary resuscitation (0.98% vs 1.35%)
				↓ Length of hospitalization (23.3 vs 28.9 days)
				↓ In-hospital deaths (15.0% vs 19.6%)
2017	Kollef, MH	Barnes-Jewish Hospital, Missouri, USA	Hospital (Medicine Ward)	Post-intervention of a rapid response system compared to pre-intervention:
				↓ Hospital mortality
				↓ Cardiopulmonary arrests per study year increment by 3.4 occurrences
				↓ Median length of stay per study year increment by 0.08 days
2020	Monteith, M	Hamilton Health Sciences, Ontario, Canada	Hospital (Acute Care Facilities)	↓ Code blues called by 11% following intervention of an early warning system
2021	Na, SJ	Samsung Medical Centre, South Korea	Hospital (General Ward)	Post-intervention of an automated alert and activation system for medical emergency teams compared to pre-intervention:
				↓ Time from deterioration to emergency medical team activation from 60 to 34 min
				↓ Unplanned ICU admission rates from 71.8% to 41.2%
				↓ Hospital mortality from 38.5% to 27.2%
2011	Sawyer, AM	Barnes-Jewish Hospital, Missouri, USA	Hospital (Medicine Ward)	Post-intervention of an automated sepsis screening and alert system compared to pre-intervention:
				↑ Number of patients receiving > 1 interventions by 15%
				↑ Antibiotic escalation from 23.8% to 36.0%
				↑ IV fluid administration from 23.8% to 38.2%
				↑ Oxygen therapy from 8.3% to 20.2%
				↑ Microbiologic cultures and radiographic imaging
2017	Subbe, CP	Ysbyty Gwynedd Hospital, UK	Hospital (General Medicine Ward)	Post-intervention of an automated vital signs monitoring and notification system compared to pre-intervention:
				↑ Number of patients with DND order
				↓ Cardiac arrests from 14 to 2 events
				↓ Mortality from 173 to 147 patients
2015	Umscheid, CA	University of Pennsylvania Health System, Pennsylvania, USA	Hospital (Non-Critical Care Services)	Post-intervention of an automated sepsis early warning and response system compared to pre-intervention:
				↓ Sepsis alert triggers for at-risk patients from 3.8% to 3.5%
				↑ Early sepsis care, ICU transfer, system activations for general medicine units
				↓ Trend in hospital mortality
				↑ Trend in discharge from hospital
2021	You, SH	Seoul National University Hospital	Hospital (Surgical Ward)	Post-intervention of an automated real-time alerting system compared to pre-intervention:
				↑ Medical emergency team alert activations from 14.4 to 26.3 per 1000 admissions
				↓ In-hospital mortality from 15.1 to 12.9 per 1000 admissions
2014	Young, RS	Northwestern Memorial Hospital, Illinois, USA	Hospital (Hematology-Oncology Units)	↓ Number of codes per 100 unit discharges by 50% following intervention of a modified early warning score
Command Center				
2017	Chan, Carri	Johns Hopkins Hospital, Baltimore, USA	Hospital	Post-intervention of a command center compared to pre-intervention:
				↓ ED patients waiting for bed by 30%
				↓ Time to retrieve data and identify patients for transfer by 1 h
				↑ Occupancy from 85% to 92%
				↓ Boarding of ED patients to medicine beds from 9.7 to 6.3 h
2022	Collins, BE	Humber River Hospital, Ontario, Canada	Hospital	Harm score at HRH using a command center compared to all Ontario-based hospitals per 100 patients:
				↓ Harm score overall (2.2 vs 5.7)
				↓ Harm score for medication conditions (1.0 vs 3.3)
				↓ Harm score for infection conditions (0.5 vs 1.9)
				↓ Harm score for patient accidents (0.1 vs 0.2)
				↓ Harm score for associated procedures (0.8 vs 1.3)
2018	Davenport, PB	Carilion Clinic, Virginia, USA	Hospital (Trauma and Emergency Care)	Post-intervention of a centralized operations center compared to pre-intervention:
				↑ Patient transfer volumes by 19%
				↑ ED admission volume by 7%
				↓ ICU patient length of stay by 0.3 days
2016	Lovett, PB	Jefferson University Hospital, Pennsylvania, USA	Hospital	Post-intervention of a centralized Patient Flow Management Centre compared to pre-intervention:
				↑ Total admissions per month from 2677 to 2810 patients
				↑ ED visits per month from 4850 to 5224 visits
				↑ Completed patient transports per month 11,475 to 13,967 patients
				↑ Mean patient transport time from 35 to 36 min
				↓ Ambulance diversion per month from 86 to 7 h
				↓ ED visits without medical team examination by 2.5%
				↓ Median ED door to provider time from 74 to 41 min
				↓ Mean EVS response time from 77 to 32 min
				↓ Mean EVS turn time from 115 to 72 min
				↓ Mean bed request to assign time from 153 to 105 min

## Author contributions

**Conceptualization:** Liza Grosman-Rimon, Pete Wegier, Barabra E. Collins.

**Visualization:** Liza Grosman-Rimon, Donny H.Y. Li.

**Writing – original draft:** Liza Grosman-Rimon.

**Writing – review & editing:** Liza Grosman-Rimon, Pete Wegier, Barabra E. Collins.
